# Integrated Deterministic and Probabilistic Methods Reveal Heavy Metal-Induced Health Risks in Guizhou, China

**DOI:** 10.3390/toxics13060515

**Published:** 2025-06-19

**Authors:** Qinju Li, Dashuan Li, Zelan Wang, Dali Sun, Ting Zhang, Qinghai Zhang

**Affiliations:** School of Public Health, Guizhou Medical University, Guiyang 561113, China; leeqinju@163.com (Q.L.); lidashuan1997@163.com (D.L.); wzl2010kaoyan@163.com (Z.W.); dalisun11@163.com (D.S.)

**Keywords:** heavy metals, agricultural soil, health risk assessments, target organ toxic dose method

## Abstract

Due to high geological background and intensive mining activities, soils are prone to heavy metals (HMs) accumulation and ecological fragility in Guizhou Province, China. A total of 740 topsoil samples were therefore collected, and aimed to determine the concentrations of As, Cd, Cr, Hg, and Pb, estimate the ecological pollution, and evaluate the carcinogenic and non-carcinogenic health risks to humans. Results showed As (1.08%) and Cd (24.46%) in soil exceeded standards. The Igeo showed that Cr (1.49%) and Hg (31.62%) in soil were at light pollution levels; single factor pollution index (PI) showed that Cd (21.35%) in soil was mildly polluted; risk index (RI) as at a low risk level. Notably, both deterministic and Monte Carlo analyses revealed unacceptable carcinogenic risks for As and Cr in children, with traditional methods potentially underestimating As risks. Moreover, Target-Organ Toxicity Dose (TTD) revealed soil HMs as a higher risk to hematological health, with notable health risks posed by Pb in children. It is noted that spatial distribution analysis suggested that the southwestern region of Guizhou Province should be prioritized for health risk management and control. By integrating the uniqueness of geological environments, multi-dimensional health risk assessments, and spatial distributions, the present study provides a scientific basis for assessing HMs pollution risks and soil health risks in the karst regions.

## 1. Introduction

Heavy metals (HMs) contamination in soil emerged as a critical environmental problem due to their inherent toxicity, persistent retention, non-degradability, and sustained bioavailability [1,2]. Soil is an important carrier of HMs. Excessive HMs can disrupt the productivity and quality of soil when entering into the soil layer [3]. Furthermore, severely contaminated soils may serve as a persistent source of groundwater and ecosystem contamination [4]. More seriously, when the concentration of HMs such as lead (Pb), arsenic (As), and mercury (Hg) in the farmland soil reaches a certain level, it will destroy the internal balance of the farmland [5]. It is important to note that soil ecosystems, particularly those of farmland, are crucial for human survival and development [6]. It is essential to evaluate the contamination status and ecological risks associated with HMs contamination in agroecosystems.

Soil contamination by HMs varies across China, with southern provinces bearing the brunt of the problem; these regions should be considered key areas for monitoring and managing HM pollution. Simultaneously, cadmium (Cd), Hg, Pb, chromium (Cr), and arsenic (As) have been identified as the main HMs requiring targeted control efforts within Chinese soils [7]. Therefore, this study focuses on these five HMs. In, addition, Southwest China constitutes a pivotal component of karst region all over the world [8]. Karst area is a fragile ecosystem prone to human impacts with high geochemical background value and limited HM capacity. Karst landscapes, formed from carbonate rock formations, are among the regions naturally characterized by elevated HM concentrations due to their unique geological composition. Research has shown that as these carbonate-rich areas undergo weathering, essential elements like calcium and magnesium leach out, leading to either the preservation or further accumulation of HMs. HMs typically accumulate in the residue, showing a steady proportional rise in both concentration and volume. This persistent buildup allows them to remain detectable in regions with minimal bedrock composition, even following extensive soil formation processes [9,10]. Geological surveys reveal that carbonate rock formations in Guizhou Province cover approximately 1.1 × 10^5^ km^2^, which accounts for 73% of the provincial territory [11] and ranks first in China. And, the researches reported that mining and smelting in Southwest China could release HMs to cause superimposed soil pollution [12]. Presently, these techniques of single factor pollution index (PI), Nemero pollution index (P_N_), geo-accumulation index (Igeo), ecological risk (Er) and risk index (RI) were all conducted to investigate the ecological risks polluted by HMs in karst areas [13,14]. Tang et al. [15] found that P_N_ was moderately polluted for the agricultural soils of karst areas. Qin et al. [16] indicated that Er in the karst area of Yunnan Province reached moderate risk accounting for 55.27% of the total samples. Therefore, the ecological pollution status of HMs in the karst areas deserves further attention.

The health risk assessment, a quantitative approach, evaluates potential human exposure risks through ingestion, dermal contact, and inhalation [17,18]. Specifically, the concentration of HMs and exposure parameters were the main considerations for deterministic risk assessment [19,20]. However, the deterministic risk, calculated by fixed values, relies on the actual magnitude of the risk defined by individual differences, age, physical condition, gender, and metabolic parameters [21,22]. In contrast, probabilistic risk simulation provided a more accurate basis for risk management and remediation [23]. Based on probabilistic modeling, the Monte Carlo simulation technique incorporates the variability in critical exposure traits, such as the fluctuation in soil ingestion rate (R_ing_), body weight (BW) and exposure frequency (EF). In probabilistic risk assessment, every variable and parameter is treated as a probability distribution rather than a fixed value. This approach dramatically minimizes the uncertainties inherent in health risk evaluations. The Monte Carlo simulation method follows these essential steps: First, probability distributions are established for HM concentration parameters. Next, uncertain parameter values are generated according to these distributions. The model then performs 10,000 randomized samplings across these parameter ranges, feeding them into the risk assessment calculations. Finally, the simulated output parameters are analyzed to generate cumulative probability distributions that quantify potential health risks [24,25]. Therefore, Monte Carlo simulation can well make up for the deficiencies of classical techniques. This technique can estimate the probability of pollutants exceeding the danger threshold and prioritize the part of health risk control, effectively conducting probabilistic health risk analysis [26]. For example, Eslami et al. [27] studied the health risks of pesticides on fruits, and the Monte Carlo simulation they adopted revealed that the total hazard quotient (THQ = 36.7%) of children was significantly higher than that of adults (7.8%). Traditional mean analysis was unable to capture this difference, demonstrating the precise identification ability of probability methods for sensitive populations. The emphasis suggested that evaluating probabilistic risk could yield a more suitable health risk assessment, to some degree [28]. In addition, the Target-Organ Toxicity Dose (TTD), a method for risk characterization of specific toxicological endpoints, which is an improvement on the traditional health risk assessment. It not only accounts for the critical effects of pollutants but also integrates the assessment of toxic doses across multiple target organs for diverse HMs, thereby significantly enhancing the precision of risk evaluations. To some extent, it places a particular emphasis on the potential impacts on target organs when pollutant concentrations surpass critical exposure doses [29]. Still, it is limited to establishing a more comprehensive and multi-perspective assessment on integrating deterministic risk, probabilistic risk and target organ toxicity dose to pay attention to human health.

As the discussion above, our study aimed to (1) systematically evaluate the spatial distribution and contamination severity of HMs; (2) assess the carcinogenic and non-carcinogenic health risks of HMs using a deterministic assessment and the Monte Carlo method; and (3) estimate the non-carcinogenic health risks of HMs using the TTD method. The present study can provide a theoretical framework for the scientific evaluation and human health risk assessment of HMs contamination in karst areas of Guizhou Province.

## 2. Materials and Methods

### 2.1. Study Area

Guizhou Province (24°37′ N–29°13′ N, 103°36′ E–109°35′ E), lies within the eastern segment of the Yunnan-Guizhou Plateau, featuring elevated ground to the west and descending terrain to the east. The annual temperatures hover between 14–16 °C and rainfall typically ranges from 1100–1400 mm. Spanning 176,167 square kilometers, this vital agricultural hub supports a population of around 35.81 million people and plays a key role in China’s grain production [30].

### 2.2. Sample Collection and Analysis

**Figure 1:**
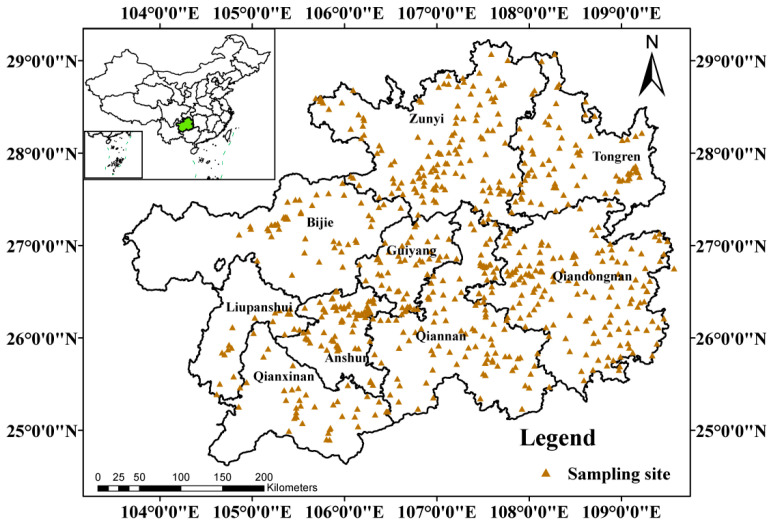
Location of the study area and distribution of sampling sites.

In autumn 2017, 740 topsoil samples (from 0–20 cm depth) were gathered from Guizhou’s farmland, nearly encompassing the entire cultivated area. The layout of sampling points referred to the Chinese national standard DZ/T 0295-2016 [31], soil samples were collected in Guiyang (GY, n = 48), Zunyi (ZY, n = 148), Anshun (AS, n = 82), Liupanshui (LPS, n = 21), Bijie (BJ, n = 51), Qiandongnan (QDN, n = 151), Qiannan (QN, n = 119), Qianxinan (QXN, n = 44) and Tongren (TR, n = 75). The distributions of sampling sites were shown in Figure 1.

To ensure a representative composite sample, five individual subsamples were gathered within a roughly 10-m radius of the target site and carefully homogenized. Following collection, all specimens were left to air-dry under ambient laboratory conditions. The soil sample processing procedure was conducted as follows: First, a 2 mm sieve was employed to remove animal residues, stones, and plant materials. The ground soil sample was then reduced to approximately 400 g using the quartering method. Subsequently, the entire sample was uniformly sieved through a 0.25 mm sieve. Of the resulting material, one-quarter was allocated for soil pH measurement, while the remaining portion (300 g) was further sieved through a 0.15 mm sieve for the determination of HMs in the soil [32]. Subsequently, 0.25 g of soil was transferred into a Teflon crucible, and then a 10 mL mixture of nitric acid and perchloric acid in a 4:1 ratio, along with 2 mL of hydrofluoric acid, was added. The crucible was subsequently heated to promote digestion. Once digested, the solution was moved to a 25 mL colorimetric tube and topped up with ultra-pure water to the required volume. The Pb, Cd, and Cr contents in the soil were then measured using Inductively Coupled Plasma-Mass Spectrometry (ICP-MS) [33]. ICP-MS allows for simultaneous multi-element detection, offering rapid, highly sensitive analysis of trace amounts [34]. In another process, about 0.3 g of soil sample via a 0.15 mm sieve was weighed and placed into a 50 mL colorimetric tube. The sample was slightly wetted with water and then mixed with 10 mL of a 1:1 aqua regia solution (HCl-HNO_3_-H_2_O, 3:1:4). This mixture was digested in a boiling water bath for 2 h, then allowing to cool, and diluting with ultra-pure water to the desired volume. The Hg content was measured using an Atomic Fluorescence Spectrometer (AF-630A) [35]. For As determination, the aliquot was taken and mixed with thiourea and ascorbic acid [36]. Hg and As were detected by AFS, and this method has high sensitivity and accuracy [37].

To measure the pH of soils, about 10 g of soil were carefully weighed and transferred into a 50 mL beaker. Then, 25 mL of water was poured in to eliminate any trapped carbon dioxide. The mixture was stirred vigorously to ensure thorough blending and left to sit for half an hour. Finally, the pH level was measured using a glass electrode.

During the determination process, GBW07408 (from the National Standards Research Center of China) was utilized for HM content analysis and quality control. The recovery rate of spiked samples was maintained between 90% and 110%. Parallel samples were taken every 30 samples, with relative deviations kept within 10%.

### 2.3. Contamination Assessment

The PI, P_N_ and Igeo were applied to assess the contamination of HMs in farmland in Guizhou Province. The PI assessed individual HM pollutant concentrations in the soils [38], while the P_N_ gauged the overall pollution impact of multiple HMs [39]. These indices are calculated as follows:

(1) $$PI=\frac{C_{i}}{S_{i}}$$

(2) $$P_{N}=\sqrt{\frac{{PI}_{ave}^{2}+{PI}_{max}^{2}}{2}}$$

where *C_i_* is the measured concentration of HM *i*, *S_i_* is the standard evaluation value, which is the soil pollution risk screening value in the “Soil environmental quality standard” [40]. The pollution level classifications for PI and P_N_ were shown in Table S1 [41].

Considering the impact of natural diagenesis on the background values, the geological accumulation index (Igeo) was employed to assess the contamination of HMs, and it identified the influence of anthropogenic activities [42]. It was calculated as follows:

(3) $$I_{geo}=\log_{2}\left[\frac{C_{i}}{\left(1.5\times B_{i}\right)}\right]$$

where *B_i_* is the geochemical background value of HM i in the local soil [43], *C_i_* is the measured concentration of HM *i*, and 1.5 is the coefficient of variation that results from rock formation [44]. The pollution level classification for Igeo was shown in Table S2 [42].

### 2.4. Potential Ecological Risk Assessment

The potential ecological risk index method proposed by Hakanson [38] can comprehensively consider the ecotoxicity of pollutants and ecological environmental factors. This approach effectively captures the overall influence of different contaminants on the ecosystem. This index can be used to quantitatively analyze and predict potential ecological risks. RI is the sum of the ecological health risk index for each HM (Er), calculated as follows:

(4) $$E_{r}=T_{i}\times \left(\frac{C_{i}}{S_{i}}\right)$$

(5) $$RI=\sum E_{r}$$

where *T_i_* is the toxic response factor (As: 10, Cd: 30, Cr: 2, Hg: 40, Pb: 5). The risk levels classifications for Er and RI were shown in Table S3 [38].

### 2.5. Health Risk Assessment

The health risk assessment recommended by the USEPA for human exposure to HM was quantified both non-carcinogenic and carcinogenic risks via oral ingestion (ing), inhalation (inh) and dermal contact (dermal), respectively [45]:

(6) $${ADD}_{ing}=C_{S}\times \frac{R_{ing}\times EF\times ED}{BW\times AT}\times 10^{-6}$$

(7) $${ADD}_{inh}=C_{S}\times \frac{R_{inh}\times EF\times ED}{PEF\times BW\times AT}$$

(8) $${ADD}_{der}=C_{S}\times \frac{AF\times SA\times ABS\times EF\times ED}{BW\times AT}\times 10^{-6}$$

where ADD_ing_, ADD_inh_, and ADD_der_ represent the average daily doses of HMs in the soil in mg/(kg·d); *C_S_* is the soil HM concentration (mg/kg). The interpretation and values of the exposure parameters are shown in Table S4 [46,47,48].

The formulas below were used to compute the non-carcinogenic and carcinogenic risk indices:

(9) $${HI}_{i}=\sum \frac{{ADD}_{i}}{{RfD}_{i}}$$

(10) $$THI=\sum {HI}_{i}=\sum ({HI}_{ing}+{HI}_{inh}+{HI}_{der})$$

(11) $${CR}_{i}=\sum \left({ADD}_{i}\times SF\right)$$

(12) $$TCR=\sum {CR}_{i}$$

HI represents the non-cancerous health risk associated with a single HM across various exposure routes, while the THI aggregates the risks from multiple HMs. If either the THI or HI exceeds 1, it signals a possible risk of health problems [49]. *RfD_i_* indicates the non-carcinogenic average daily reference dose for HM i. CR is the carcinogenic risk factor for all exposure pathways for a single HM, the TCR indicates the total carcinogenic risk for multiple HMs. SF is a carcinogenic slope factor. When the CR values surpass the risk cutoff of 1 × 10^−4^, it suggests that humans face significant risks of cancer. Conversely, if the CR values fall below the commonly accepted threshold of 1 × 10^−6^, they are typically viewed as posing an insignificant threat to human health [50]. The values of exposure parameters relevant to adults and children in the health risk assessment are shown in Table S5 [46,47,48,51].

To address uncertainties and variability in risk quantification, a probabilistic framework employing Monte Carlo simulations was implemented. Computational analyses were conducted using Oracle Crystal Ball, with 10,000 iterative samplings at a 95% confidence interval, drawing stochastically from predefined exposure parameter distributions [52]. This approach generated probabilistic health risk profiles, while the parameter configurations for probability density functions in the risk assessment model [53] were detailed in Table S6 [46,54,55,56].

In addition, TTD is an improvement on the HI method, and the toxic dose of HM in multiple target organs is included in the evaluation scope, which can more accurately reflect the specific health risks of pollutants to humans [57]. At present, the corresponding target organ toxicity data for Cd, Pb, As and Cr are relatively complete, and the corresponding target organ toxicity effect endpoint data have been reported, while the target organ toxicity data for Hg are relatively lacking [58]. The formula for calculating HI in the TTD method is as follows:

(13) $${HI}_{TTD}=\sum \frac{{ADD}_{i}}{{TTD}_{i}}$$

(14) $${THI}_{TTD}=\sum {HI}_{i}$$

where the TTD_i_ value is the endpoint of the toxicological effect of the corresponding target organ for each HM (Table S7) [59]. HI_TTD_ is the risk value of a single HM to the target organ. The THI_TTD_ is the sum of the HI_TTD_ of multiple HMs.

### 2.6. Data Analysis

Microsoft Office Excel 2024, IBM SPSS Statistics 27, Origin 2024 and GraphPad Prism 10 were employed for experimental data processing and analysis. Independent sample *t*-test of health risk indices (HI, CR) between adults and children were performed using Student’s *t*-test. Subsequently, Kriging interpolation of ArcGIS 10.8 software was used to conduct spatial interpolation mapping to describe the spatial distribution of HMs.

## 3. Results

### 3.1. Evaluation of Heavy Metal Pollution

The results of HMs pollution in soil were shown in Table 1. The mean values of As, Cd, Cr, Hg and Pb for the soil samples were 9.08, 0.36, 73.06,0.13 and 28.14 mg/kg, respectively. When compared with the soil background values of Guizhou Province, the exceeding rates of As, Cd, Cr, Hg and Pb were 4.46%, 5.54%, 20.95%, 62.03% and 18.92%, respectively. Furthermore, the soils in Guizhou Province mainly exhibited slight acidity, with pH values between 3.84 and 8.06 and an average of 6.14. At pH levels ≤ 5.5, between 5.5 and 6.5, and from 6.5 to 7.5, Cd exceedance rates were 37.39%, 32.03%, and 9.47%, respectively. At the pH > 7.5, the exceeding rate of As was 9.88%. The total exceedance rates of As and Cd were 1.08% and 24.46%, respectively. Moreover, the coefficient of variation (CV) was in the order of As (59.25%) > Hg (46.15%) > Cd (44.44%) > Cr (39.98%) > Pb (28.75%).

**Figure 2:**
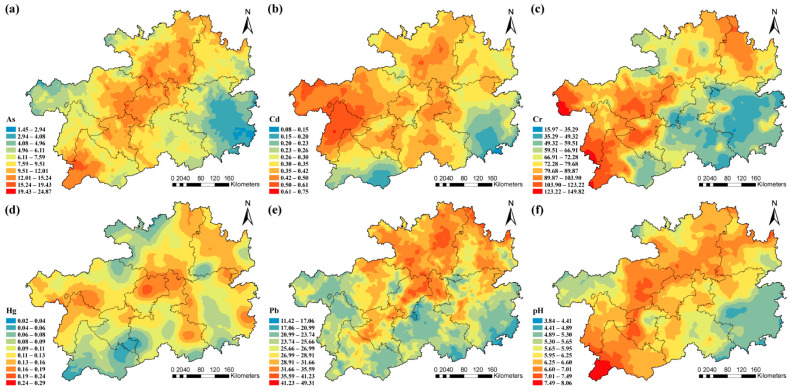
Spatial distribution of heavy metal pollution characteristics. (**a**) As; (**b**) Cd; (**c**) Cr; (**d**) Hg; (**e**) Pb; (**f**) pH.

The kriging method was further used to analyze the current status and spatial distribution of HM contamination in agricultural soils (Figure 2). High levels of As and Hg were observed in the central area; high Cd and Cr were concentrated mainly in the western region; and Pb concentrations were higher from the northern parts, respectively. And the levels of pH were high in the southwestern part.

**Figure 3:**
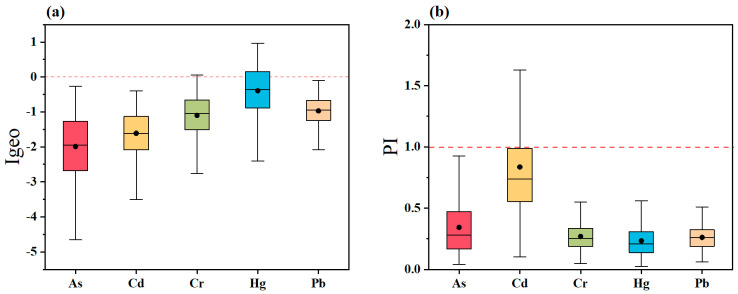
Assessment of the (**a**) Igeo and (**b**) PI.

In addition, the methods of Igeo and PI were utilized to assess the contamination status of farmland soils. assess the contamination status of farmland soils. in the Guizhou Province. The values of Igeo indicated that 1.49% of Cr and 31.62% of Hg, indicating minor pollution (Figure 3a). The average PI for HMs varied between 0.23 and 0.84. Specifically, 21.35% of the sampling sites exhibited mild Cd contamination (1 < PI ≤ 2), and 3.11% showed moderate Cd contamination (2 < PI ≤ 3) (Figure 3b). The P_N_ value was 1.77, which was at the light pollution level.

### 3.2. Ecological Risk Assessment

**Figure 4:**
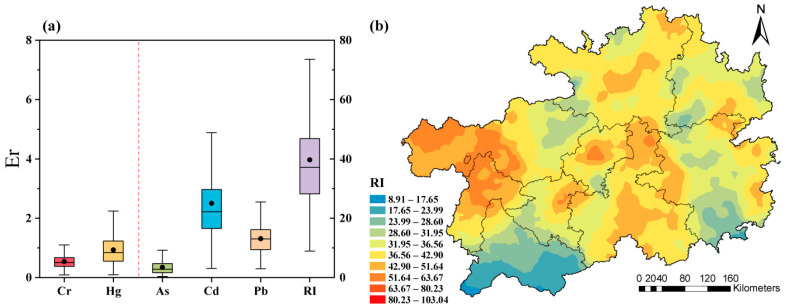
Assessment of (**a**) Er and spatial distribution of (**b**) RI.

The Er values calculated by HMs concentrations rank as the order of Cd > Pb > As > Hg > Cr, with 10.95% sites of Cd rated at a moderate risk level (Figure 4a). And, there was no ecological risk level in Guizhou because the RI value was below 80. More importantly, the spatial pattern of RI (Figure 4b) indicated higher concentrations in the western area, with slightly higher values in the central region.

### 3.3. Deterministic Risk Assessment

The results of the deterministic risk assessment were shown in Table 2. The results for the three exposure pathways were in the order of HI_ing_(1.12 × 10^−1^) > HI_dermal_(1.54 × 10^−2^) > HI_inh_(7.62 × 10^−4^) in adults and HI_ing_(7.54 × 10^−1^) > HI_dermal_(7.22 × 10^−2^) > HI_inh_(1.32 × 10^−3^) in children, indicating that oral ingestion is the primary exposure pathway for non-carcinogenic risks. The deterministic risks posed by HMs, the order of HI was Cr(5.12 × 10^−2^) > As(4.59 × 10^−2^) > Pb(3.03 × 10^−2^) > Hg(6.82 × 10^−4^) > Cd(6.42 × 10^−4^) in adults and Cr(3.12 × 10^−1^) > As(3.05 × 10^−1^) > Pb(2.02 × 10^−1^) > Hg(4.50 × 10^−3^) > Cd(4.03 × 10^−3^) in children. Although all soil HI values were under 1, 27.84% THI values in children and 30% TCR values in adults were above the acceptable range. At the same time, the order for CR was Cr(6.32 × 10^−5^) > As(2.04 × 10^−5^) > Cd(3.27 × 10^−6^) > Pb(3.56 × 10^−7^) in adults and Cr(4.06 × 10^−4^) > As(1.36 × 10^−4^) > Cd(2.19 × 10^−5^) > Pb(2.39 × 10^−6^) in children. CR(Cr) exceeded the acceptable range in 9.59% of adults. Specifically, the CRing values of As and Cr for children in the ingestion pathway were greater than 1 × 10^−4^. And, the exceeding rate of CR(As) in children was 57.30%. The Student’s t-test showed that there were significant differences in all health risk indicators between children and adults (Figures S1 and S2). For THI, TCR, As, Cd, Cr, Hg and Pb, the non-carcinogenic and carcinogenic risks were notably greater in children compared to adults (*p* < 0.0001).

**Figure 5:**
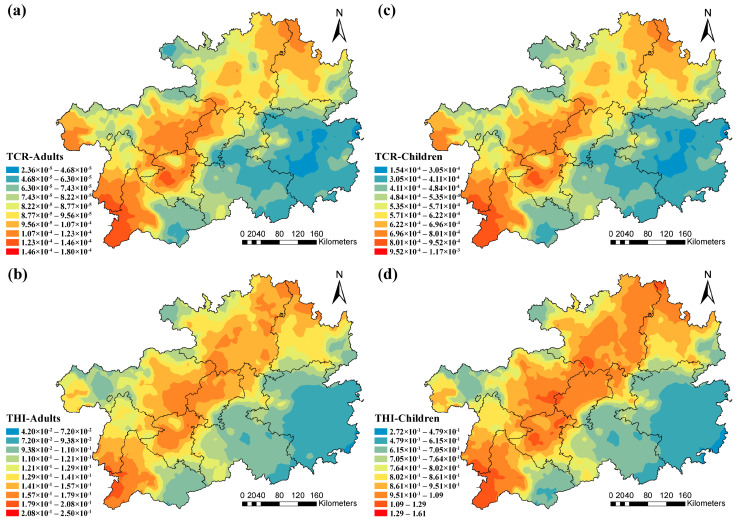
Spatial distribution of total carcinogenic and non-carcinogenic risks in (**a**,**b**) adults and (**c**,**d**) children.

The kriging technique was further employed to map out the spatial patterns of THI and TCR among both adults and children (Figure 5). The high TCR values of adults and children were primarily located in the southwest and central parts areas (Figure 5a,c), and the high THI values were mainly distributed in the southwest, central and northeast areas (Figure 5b,d).

### 3.4. Probabilistic Risk Assessment

**Figure 6:**
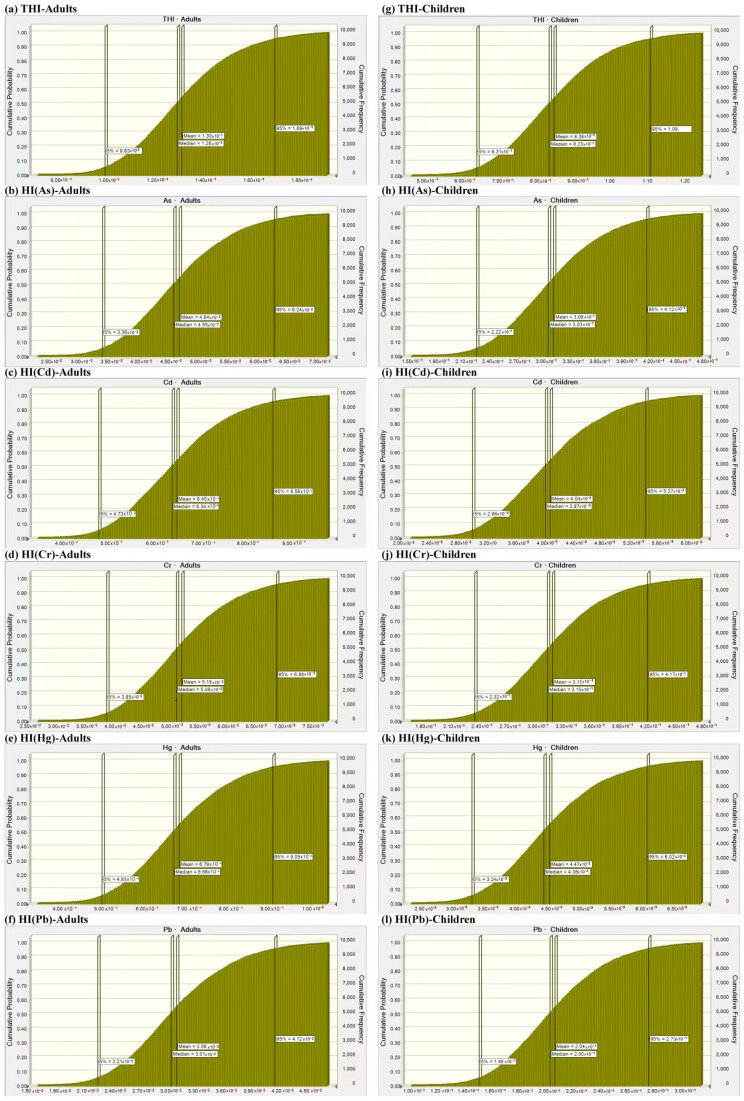
Probabilistic non-carcinogenic risk assessment of heavy metals in (**a**–**f**) adults and (**g**–**l**) children.

**Figure 7:**
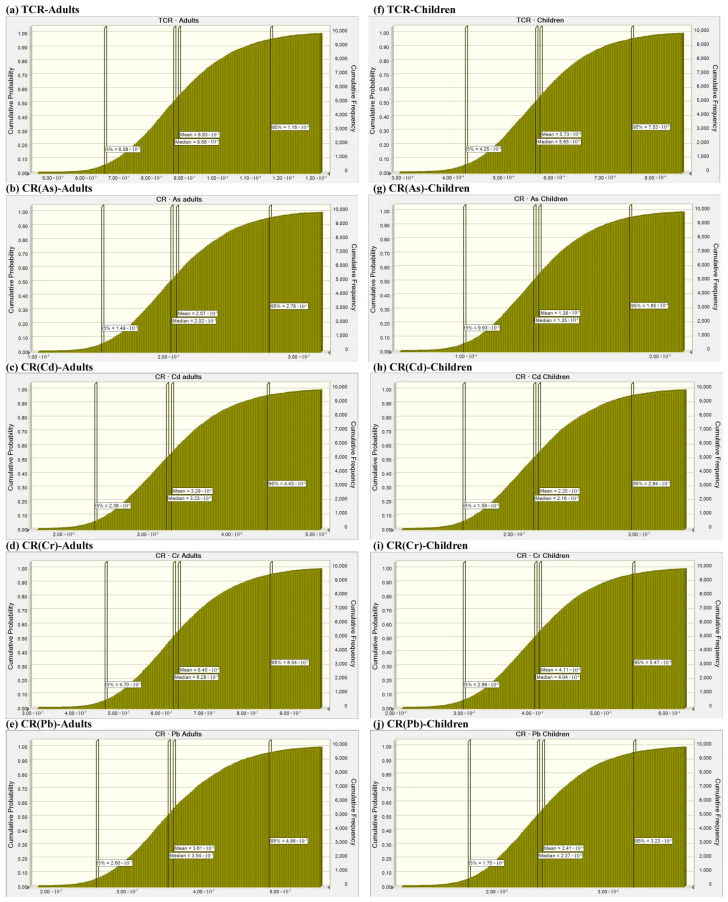
Probabilistic carcinogenic risk assessment of heavy metals in (**a**–**e**) adults and (**f**–**j**) children.

The probabilistic assessment by Monte Carlo simulation was shown in Figure 6 and Figure 7. The probabilistic risks ranked as follows: for mean HI values, Cr(5.18 × 10^−2^) > As(4.64 × 10^−2^) > Pb(3.06 × 10^−2^) > Hg(6.79 × 10^−4^) > Cd(6.45 × 10^−4^) in adults (Figure 6b–f) and Cr(3.15 × 10^−1^) > As(3.08 × 10^−1^) > Pb(2.04 × 10^−1^) > Hg(4.47 × 10^−3^) > Cd(4.04 × 10^−3^) in children (Figure 6h–l); similarly, for mean CR values, the order was Cr(6.40 × 10^−5^) > As(2.07 × 10^−5^) > Cd(3.29 × 10^−6^) > Pb(3.61 × 10^−7^) in adults (Figure 7b–e) and the order was Cr(4.11 × 10^−4^) > As(1.38 × 10^−4^) > Cd(2.20 × 10^−5^) > Pb(2.41 × 10^−6^) in children (Figure 7g–j). Obviously, the Cr values both in CR and HI were the highest among all of the investigated HMs. In adults, HI(Cr) contributed 39.85% to THI, followed by HI(As) with 35.69%. In children, HI(Cr) contributed 37.68% to THI, followed by HI(As) with 36.84%. In children, there was an 11.67% probability that the THI value exceeded the exposure risk value. The acceptable threshold for CR(Cr) was likely to be exceeded in adults, but it was guaranteed to be exceeded in children, with a 100% probability. Additionally, there was a 94.62% probability that the acceptable threshold for CR(As) would be exceeded in children. The total probabilistic carcinogenic risk was 20.69% for adults and 100% for children, respectively. The Student’s t-test showed that there were significant differences in all health risk indicators between children and adults (Figures S3 and S4). For THI, TCR, As, Cd, Cr, Hg and Pb, the probabilistic non-carcinogenic and carcinogenic risks were notably greater in children compared to adults (*p* < 0.0001).

### 3.5. Health Risk Assessment Based on the TTD Method

**Figure 8:**
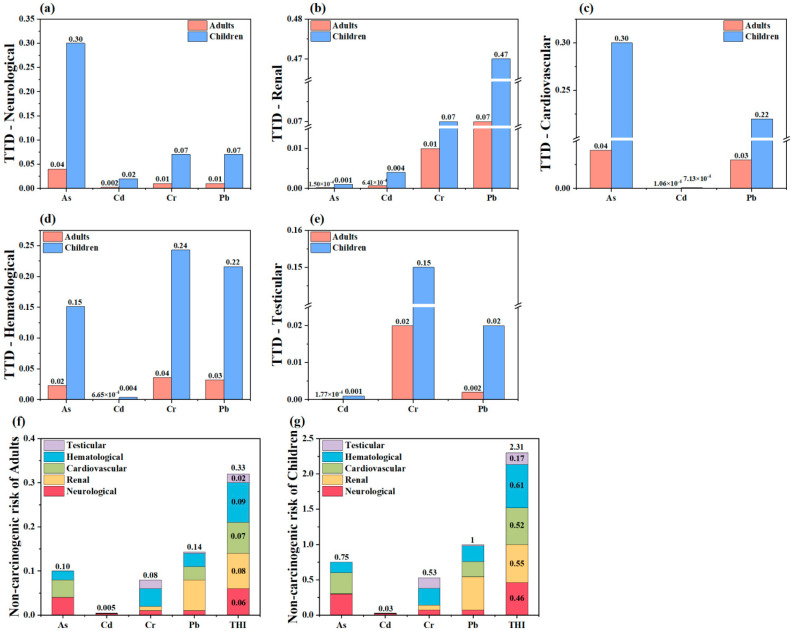
Non-carcinogenic risk of (**a**–**e**) different target organs and (**f**,**g**) different populations based on TTD.

**Figure 9:**
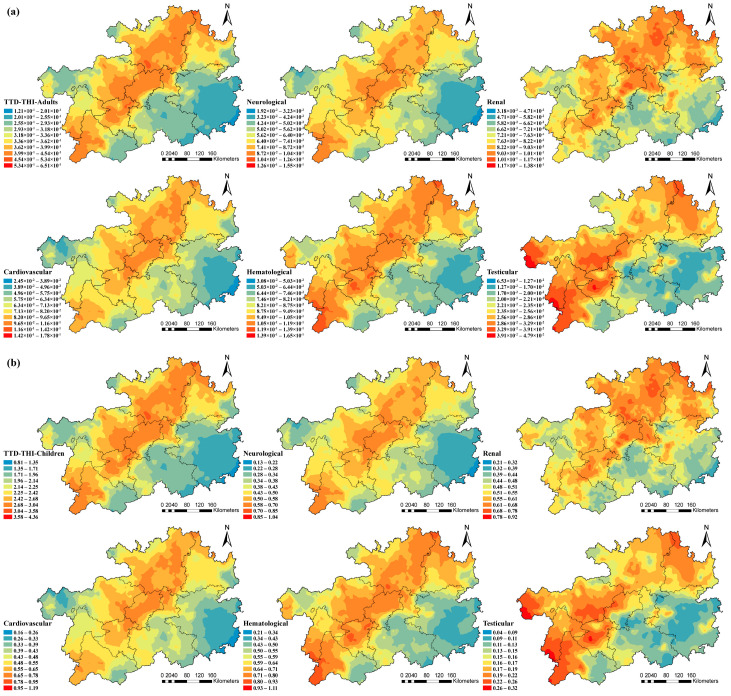
Spatial distribution of total non-carcinogenic risks of different target organs in adults (**a**) and children (**b**).

Oral ingestion, as the primary exposure way of health risk, was selected as a non-carcinogenic risk assessment modified by the TTD. From the target organs, the cumulative risks in our study were 0.09, 0.08, 0.07, 0.06 and 0.2 in adults (Figure 8f) and 0.61, 0.55, 0.52, 0.46 and 0.17 in children (Figure 8g), respectively. It showed that HI_TTD_(Hematological) had the highest contribution rate to THI_TTD_, which was 27.27% and 26.41% in adults and children, respectively. From the perspective, the Pb, As, Cr and Cd values for cumulative risks were 0.14, 0.10, 0.08 and 0.005 in adults and 1, 0.75, 0.53, and 0.03 in children, respectively. The data showed that the contributions of HI_TTD_(Pb) were 42.42% in adults and 43.29% in children. In total, The THI_TTD_ was 2.56 times higher than the definitive risk assessment of THI_Adults_ and 2.79 times that of THI_Children_. The health risks of HMs in different target organs were illustrated in Figure 8. The neurological and cardiovascular systems were most sensitive to As (Figure 8a,c), the renal system to Pb (Figure 9b), and the hematological and testicular systems to Cr (Figure 8d,e). In addition, children faced greater health risks compared to adults.

The spatial pattern of HI_TTD_ was uniformly comparable across adults (Figure 9a) and children (Figure 9b) for various target organs. The high-risk areas for the neurological and cardiovascular systems were primarily in the central region, those for the renal system in the northern, and those for the testicular system in the west. The high values of THI_TTD_ were primarily located in the southwest, central and northeast regions.

## 4. Discussion

### 4.1. Heavy Metals Pollution Analysis

In most cases, the adsorption of metal elements onto soil particle surfaces intensifies as soil pH levels increase [60]. We found that the higher Cd concentrations of samples are higher at the low pH. To some extent, it might be contributed to the low solubility of soil Cd at a high pH accounting with the properties of calcareous soil [61]. In contrast, the present study indicated that As exceeded the limit at high pH levels. As previously study described, the solubility of As in soil is possibly rising with the soil pH increasing [62]. Furthermore, the CV reflected the variability and dispersion of soil elements. Elements with high CV may be affected by human activities [63]. As, Cd, Cr and Hg showed high variability (CV ≥ 36%), indicating that they had high spatial heterogeneity [64], which may be due to the impacts of parent rocks and the process of soil formation [16].

The PI of Cd was the highest, primarily due to its relatively high toxicological response factor [65]. Investigation of the agricultural soil near the mining area in central Guizhou showed that the P_N_ was 2.5 [14], which was higher than the result of this study, as it was near the mining area possibly. Although the PI for other HMs were within the standard limits, 24.46% of the sites showed Cd contamination. Therefore, the P_N_ was 1.77, indicating that Guizhou province was under slight pollution. The P_N_ describes the possibility of pollution, the risk amount of the indicated pollution and it is also able to measure the reach of HMs pollution to the topsoil level, taking into account the risks of all referenced HMs [66]. The Igeo index considers the effects of natural diagenesis and anthropogenic activities [67], and the Igeo of 31.62% of Hg was slightly polluted, indicating that Hg was affected by anthropogenic activities. Guizhou is one of the major Hg-producing regions in China, consistently ranking first in terms of Hg ore reserves and production. The related mining and smelting activities will produce a large amount of Hg-containing waste gas, waste water and waste residue, which is very likely to lead to HMs contamination of its neighboring soils [68]. Therefore, the contamination of Hg in farmland soil should be noted within the Guizhou Province. Moreover, the Er of 10.95% Cd indicated a moderate pollution level. Although the results of RI indicated that the study area was at a low risk level, the impact of Cd should still be taken seriously. The Igeo focuses on quantifying the effects of anthropogenic pollution, while other indices (such as Er and RI) pay more attention to toxicity responses or comprehensive risk assessment. Therefore, PI and Er described the pollution risk level of Cd, while Igeo indicated the impact of human activities on Hg. The pollution of Cd and Hg in the farmland soil of Guizhou Province deserves attention, suggesting that priority should be given to control and reduce their risk to the environment. The deficiency of this study is that only five HMs were investigated. However, the influence of other HMs (such as Cu, Co, Zn, etc.) is also very important. Therefore, in the subsequent research work, we consider including these several HMs in the study to assess the soil environmental quality more comprehensively.

### 4.2. Deterministic and Probabilistic Risk Assessment

Deterministic health risk assessment was used to estimate carcinogenic and non-carcinogenic risks for adults and children via ingestion, inhalation, and skin contact. Kyere et al. [69] and Demirtepe et al. [70] showed that higher HI levels were typically observed in the ingestion routes compared to inhalation and skin contact routes. This study also showed that the ingestion route contributed the most to HI. Therefore, this pathway should be valued. Similar to previous studies [71,72], our study indicated that children exhibited higher non-carcinogenic and carcinogenic risks than adults (*p* < 0.0001), primarily owing to their conduct, biological traits, and exposure duration [73]. In addition, Lu et al. [74] revealed that As and Cr contributed high CR was noteworthy, particularly in the southwestern region, which aligned with the findings of this study. The CR of Cr was higher, potentially due to its lower slope factor, posing a greater carcinogenic risk than other HMs. Meantime, there are many mineral resources in Guizhou Province with high concentrations of Cr [75]. Therefore, the health risks posed by As and Cr to children deserve attention in the southwestern region.

Monte Carlo simulation, a widely used probabilistic risk assessment technique, minimizes uncertainty and offers more comprehensive results during risk evaluation [22]. Through the evaluation of two methods, Cr had the highest risk in HI and CR, followed by As; and CR values for Cr and As exceeded the acceptable range in children. However, the mean HI and CR values of As, Cd, Cr, and Pb, for the probabilistic approach were slightly higher than those for the deterministic approach. Previous research used deterministic values to assess health risks, which eventually may underestimate the risk outcomes [76,77]. A significant reduction has been observed in exceedance probabilities for THI for children and adult TCR and CR(As) for adults relative to their safety thresholds. These phenomena suggested that the above indicators may be overestimated in deterministic assessments. Additionally, the CR of As for children exceeded the probability risk increased, among from 57.30% in deterministic assessment to 94.62%, which indicated that the risk may be underestimated. To our knowledge, deterministic and probabilistic techniques are widely used to estimate human health risks posed by various pollutants [78]. Combining the two methods to explore the health risks of HMs to people can provide a scientific foundation for policymakers to achieve risk management. A key limitation is the absence of formal sensitivity analysis. Although the main purpose of this study was to assess the overall risk probability range of soil heavy metals to the population, and Monte Carlo simulation effectively quantified the variability of the results under the uncertainty of the given parameters, this limited the identification of key risk drivers. Future studies should incorporate sensitivity and uncertainty analysis to further explain the research results in depth. In addition, Jin et al. [79] estimated the health risks of HMs in food, which gave us great enlightenment. Subsequently, we consider conducting an assessment of HMs pollution and health risks related to soil, crops and humans.

### 4.3. Health Risk Assessment Modified by the TTD Method

The health risks posed by HMs in soil to local populace were objectively evaluated by both the deterministic and probabilistic risks. However, traditional health risk assessment models only consider the most sensitive effect target organ of HMs, while actual risks arise from damage to multiple target organs simultaneously. This may result in an underestimation of the non-carcinogenic health risks posed by soil HMs contamination to humans [57,80]. Therefore, TTD was further used for risk assessment of specific target organs.

On account of Pb being more sensitive to the toxic effects of target organs, its cumulative risk value was higher than other HMs. More importantly, the HI_TTD_ value of Pb in children was 1. Therefore, the health risks caused by Pb to children should be paid attention to. In terms of a single target organ, the accumulation risk in the hematology was the highest, indicating that HMs in agricultural soil might cause damage to the hematological system of the population, although it is still within the safe threshold of soil risk. Compared with the traditional deterministic assessment results, the HI_TTD_ evaluated by the TTD model was 1.56~7.79 times than that of HI, which was mainly due to the joint action of two or more HMs in these target organs [81]. Thus, utilizing the TTD model allows for more precise measurement and comparison of cumulative risks associated with various HMs against risk values obtained through traditional methods, providing a deeper understanding of health risks to target organs [81]. To this end, the TTD addresses the limitation of the traditional method in comprehensively assessing risks across multiple target organs.

In addition, consistent with the results of traditional methods, the health risks in the southwest region are noteworthy. Due to the lack of supporting data and uneven medical resource allocation, it cannot be determined whether the incidence rates of relevant diseases in various regions of Guizhou align with research findings. Therefore, residents in high-risk areas should be mindful of undergoing regular health checks for related target organ diseases. However, none of these three health risk assessment techniques take into account the bioavailability of HMs in the soil, which may lead to an overestimation of health risks. This is the deficiency of this study. Future research will focus on the bioavailability of soil HMs and animal experiments to fully explore and verify the carcinogenic and non-carcinogenic health risks brought by related metal pollutants to the human body, to evaluate the health risks of soil HMs more comprehensively. It will facilitate decision-making regarding health risks and the formulation of appropriate public health measures.

## 5. Conclusions

Cd and Hg were the main pollutants in agricultural soil of Guizhou Province, and their distribution was affected by industrial activities and soil pH. The P_N_ indicated slight pollution in the farmland soil within the province. Children were at higher risk of non-carcinogenic and carcinogenic than adults, and the CR of Cr (3.12 × 10^−1^) and As (3.05 × 10^−1^) was particularly prominent, which exceeded the acceptable range. However, the carcinogenic risk of adults was less than 1 × 10^−4^ and did not exceed the standard. Convergent findings from deterministic modeling and Monte Carlo simulations revealed CR for As and Cr exceeding safety thresholds in children. Further, the TTD was used to assess multi-organ risk, revealing a higher risk of soil HMs for hematological health, with notable health risks posed by Pb in children. This approach addresses the limitation of the traditional method in comprehensively assessing risks across multiple target organs. It is noted that spatial distribution analysis suggested that the southwestern region of Guizhou Province should be prioritized for health risk management and control. By integrating the uniqueness of geological environments, multi-dimensional health risk assessments, and spatial distributions, this study provides a scientific basis for assessing HMs pollution risks and soil health risks in karst regions.

## Figures and Tables

**Table 1:** Concentrations of heavy metal in soils in the study area (mg/kg).

Items	As	Cd	Cr	Hg	Pb	pH
Min	1.19	0.08	15.97	0.02	11.42	3.84
Max	24.87	0.75	149.82	0.29	49.38	8.06
Mean	9.08	0.36	73.06	0.13	28.14	6.14
SD	5.38	0.16	29.21	0.06	8.09	0.96
CV%	59.25	44.44	39.98	46.15	28.75	15.64
pH ≤ 5.5	30	0.30	250	0.50	80	-
Exceeded(%) ^a^	0	37.39	0	0	0	-
5.5 < pH ≤ 6.5	30	0.4	250	0.50	100	-
Exceeded(%) ^a^	0	32.03	0	0	0	-
6.5 < pH ≤ 7.5	25	0.6	300	0.6	140	-
Exceeded(%) ^a^	0	9.47	0	0	0	-
pH > 7.5	20	0.8	350	1.0	240	-
Exceeded(%) ^a^	9.88	0	0	0	0	-
BV ^b^	20	0.66	95.9	0.1	35.2	-
Exceeded(%) ^b^	4.46	5.54	20.95	62.03	18.92	-

^a^ Soil pollution risk screening values refer to “Soil environmental quality standard” (GB15618-2018) [40]. ^b^ China National Environmental Monitoring Center (CNEMC), the Backgrounds of Soil Environment of Guizhou, China.

**Table 2:** Carcinogenic and non-carcinogenic risk assessment of heavy metals in different populations.

		Non-Carcinogenic Risks				Carcinogenic Risks			
		HI_ing_	HI_inh_	HI_dermal_	HI	CR_ing_	CR_inh_	CR_dermal_	CR
As	Adults	4.51 × 10^−2^	3.36 × 10^−4^	4.35 × 10^−4^	4.59 × 10^−2^	2.03 × 10^−5^	2.18 × 10^−8^	8.09 × 10^−8^	2.04 × 10^−5^
	Children	3.02 × 10^−1^	5.83 × 10^−4^	2.05 × 10^−3^	3.05 × 10^−1^	1.36 × 10^−4^	3.78 × 10^−8^	3.81 × 10^−7^	1.36 × 10^−4^
Cd	Adults	5.32 × 10^−4^	1.98 × 10^−5^	8.49 × 10^−5^	6.42 × 10^−4^	3.27 × 10^−6^	3.57 × 10^−10^		3.27 × 10^−6^
	Children	3.57 × 10^−3^	3.44 × 10^−5^	3.99 × 10^−4^	4.03 × 10^−3^	2.19 × 10^−5^	6.19 × 10^−10^		2.19 × 10^−5^
Cr	Adults	3.63 × 10^−2^	4.06 × 10^−4^	1.45 × 10^−2^	5.12 × 10^−2^	5.45 × 10^−5^	4.87 × 10^−9^	8.68 × 10^−6^	6.32 × 10^−5^
	Children	2.43 × 10^−1^	7.04 × 10^−4^	6.81 × 10^−2^	3.12 × 10^−1^	3.66 × 10^−4^	8.45 × 10^−9^	4.09 × 10^−5^	4.06 × 10^−4^
Hg	Adults	6.36 × 10^−4^		3.61 × 10^−5^	6.82 × 10^−4^				
	Children	4.26 × 10^−3^		1.70 × 10^−4^	4.50 × 10^−3^				
Pb	Adults	2.99 × 10^−2^		3.19 × 10^−4^	3.03 × 10^−2^	3.56 × 10^−7^	1.88 × 10^−10^		3.56 × 10^−7^
	Children	2.01 × 10^−1^		1.50 × 10^−3^	2.02 × 10^−1^	2.39 × 10^−6^	3.26 × 10^−10^		2.39 × 10^−6^
THI/TCR	Adults	1.12 × 10^−1^	7.62 × 10^−4^	1.54 × 10^−2^	1.29 × 10^−1^	7.52 × 10^−5^	2.72 × 10^−8^	8.77 × 10^−6^	8.40 × 10^−5^
	Children	7.54 × 10^−1^	1.32 × 10^−3^	7.22 × 10^−2^	8.28 × 10^−1^	5.04 × 10^−4^	4.72 × 10^−8^	4.12 × 10^−5^	5.45 × 10^−4^

## Data Availability

The data presented in this study are available on request from the corresponding author.

## Supplementary Materials

The following supporting information can be downloaded at: https://www.mdpi.com/article/10.3390/toxics13060515/s1, Figure S1. Non-carcinogenic risk significance test analysis for adults and children Note: **** *p* < 0.0001; Figure S2. Carcinogenic risk significance test analysis for adults and children. Note: **** *p* < 0.0001; Figure S3. Probabilistic non-carcinogenic risk significance test analysis for adults and children. Note: **** *p* < 0.0001; Figure S4. Probabilistic carcinogenic risk significance test analysis for adults and children. Note: **** *p* < 0.0001; Table S1: Classification of the PI and P_N_; Table S2: Classification of the Igeo; Table S3: Classification of the Er and RI; Table S4: Description of parameters in health risk assessment; Table S5: Reference dose (RfD) and cancer slope factor (SF) via oral (Ingestion), inhalation and dermal contact; Table S6: Distribution settings for each parameter in the Monte Carlo simulation; Table S7: The corresponding target organ toxicity doses for each heavy metal.
