# Supplementary material for: Integrated Deterministic and Probabilistic Methods Reveal Heavy Metal-Induced Health Risks in Guizhou, China
Source: Toxics. 2025 Jun 19;13(6):515. doi: 10.3390/toxics13060515 (PMC12197724; doi:10.3390/toxics13060515)
Supplement: Supplementary file 1 [file toxics-13-00515-s001.zip › toxics-3589559-supplementary.pdf]

Supplemental Material

Figure S1. Non-carcinogenic risk significance test analysis for adults and children
Note: ****p<0.0001

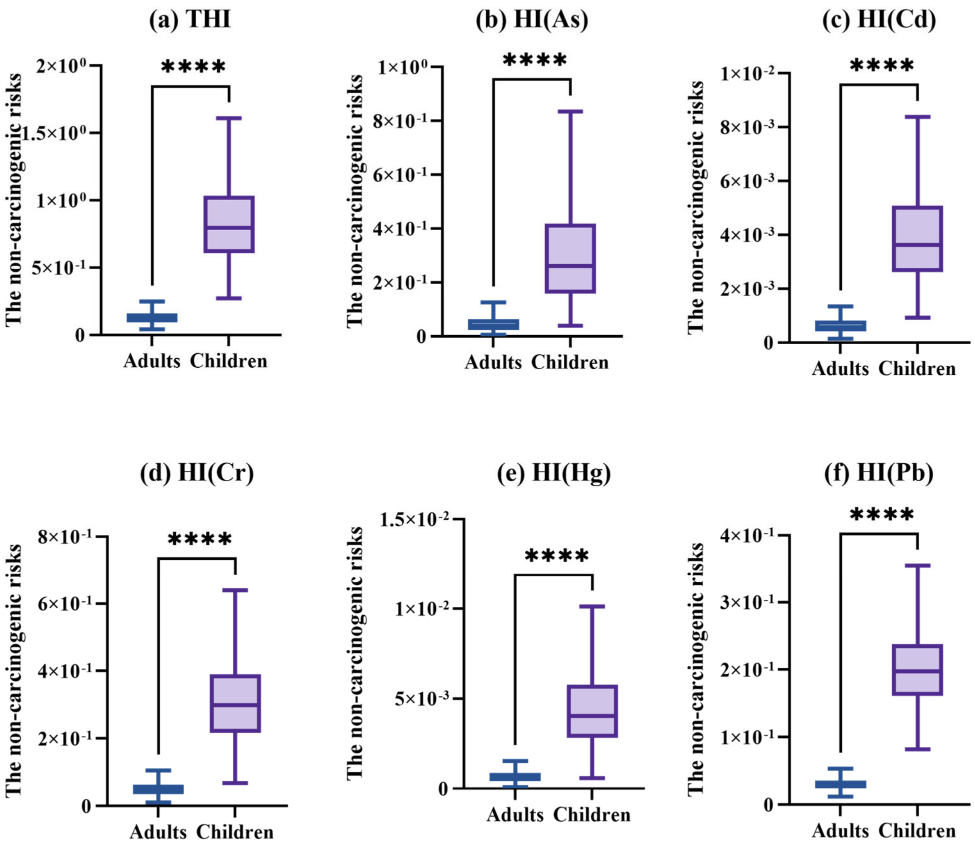

Figure S2. Carcinogenic risk significance test analysis for adults and children
Note: ****p<0.0001

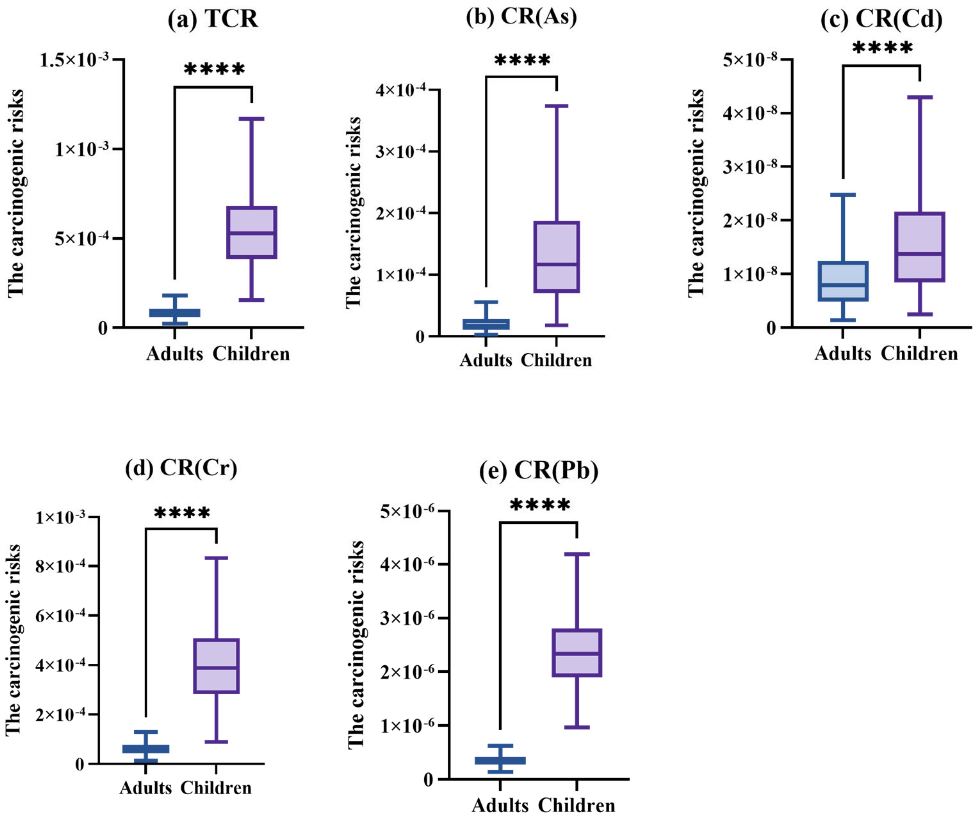

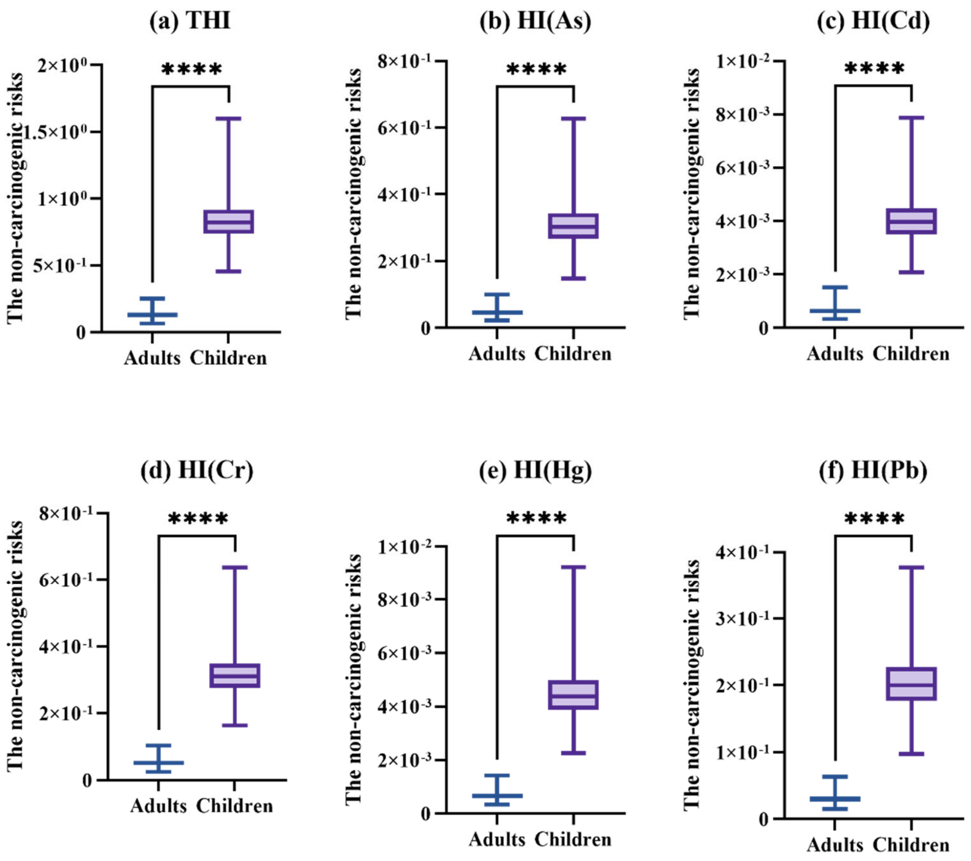

Figure S3. Probabilistic non-carcinogenic risk significance test analysis for adults and children

Note: ****p<0.0001

Figure S4. Probabilistic carcinogenic risk significance test analysis for adults and children
Note: ****p<0.0001

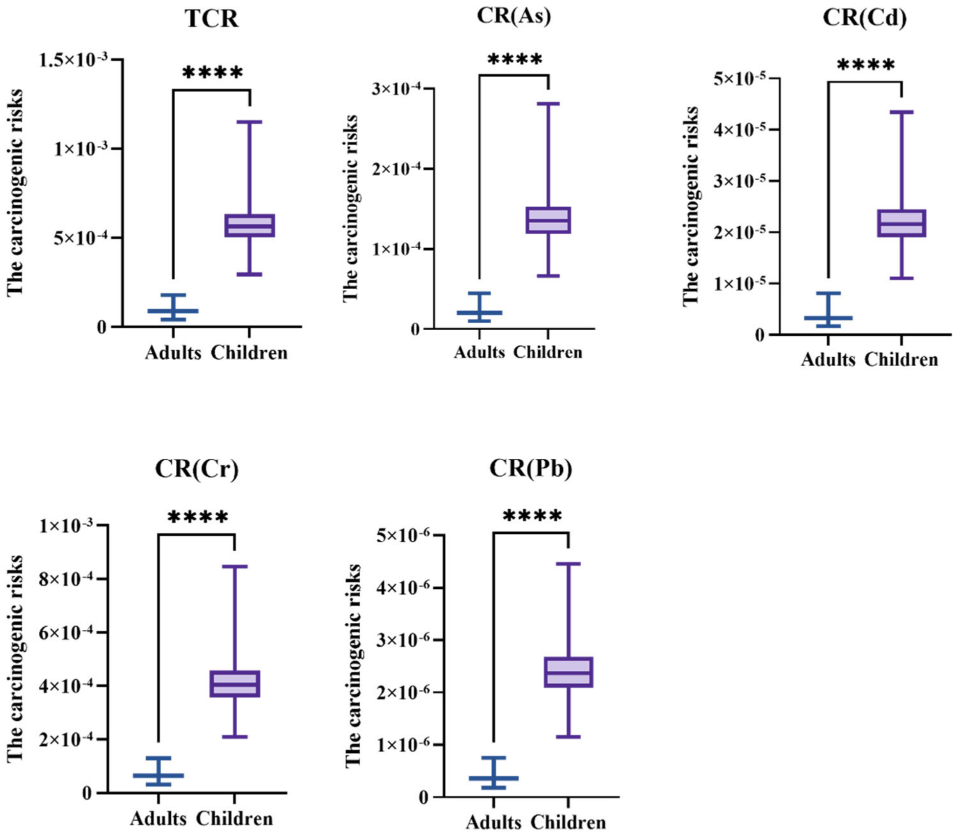

Table S1. Classification of the PI and P_N [41]

Classification	PI	Pollution Degree	P_N	Pollution Assessment
Ⅰ	$PI \leq 1$	Clean	$P_N \leq 0.7$	Clean(security)
Ⅱ	$1 < PI \leq 2$	Slight pollution	$0.7 < P_N \leq 1$	Still clean(cordon)
Ⅲ	$2 < PI \leq 3$	Moderate pollution	$1 < P_N \leq 2$	Mild pollution
Ⅳ	$PI > 3$	Severe pollution	$2 < P_N \leq 3$	Moderate pollution
Ⅴ	-	-	$P_N > 3$	Severe pollution

Table S2. Classification of the Igeo [42]

Classification	I_{geo}	Pollution Degree
0	$I_{geo} \leq 0$	Unpolluted
1	$0< I_{geo} \leq 1$	Lightly polluted
2	$1< I_{geo} \leq 2$	Moderately polluted
3	$2< I_{geo} \leq 3$	Moderately to heavily polluted
4	$3< I_{geo} \leq 4$	Heavily polluted
5	$4< I_{geo} \leq 5$	Heavily to extremely polluted
6	$I_{geo} > 5$	Extremely polluted

Table S3. Classification of the Er and RI [38]

Ecological Risk	Low	Moderate	Considerate	High	Very high
E_r	<40	40−80	80−160	160−320	>320
RI	<150	150−300	300−600	-	>600

Table S4. Description of parameters in health risk assessment [46-48]

Parameters	Description	Unit	Adults	Children
Ring	Average daily intake by ingestion	mg/d	100	200
Rinh	Inhalation rate	m^3/d	14.5	7.5
EF	Exposure frequency	d/a	350	350
ED	Exposure duration	a	24	6
BW	Average body weight	kg	61.8	19.2
AT	Average exposure time	d	9125	2190
SA	Exposed skin surface area	cm^2/d	5700	2800
AF	Adherence factor	mg/cm^2	0.07	0.2
ABS	Dermal absorption factor	%	0.1	0.1
PEF	Particle emission factors	m^3/kg	1.36×10^9	1.36×10^9

Table S5. Reference dose (RfD) and cancer slope factor (SF) via oral (Ingestion), inhalation and dermal contact [46-48,51]

Element	RfD (mg/kg/day)			SF (mg/kg/day)		
	ingestion	dermal	inhalation	ingestion	dermal	inhalation
As	3.00×10^{-4}	1.23×10^{-4}	4.29×10^{-6}	1.50	1.50	1.51×10^{1}
Cd	1.00×10^{-3}	2.50×10^{-5}	2.86×10^{-6}	6.10	-	6.30
Hg	3.00×10^{-4}	2.14×10^{-5}	-	-	-	-
Pb	1.40×10^{-3}	5.24×10^{-4}	-	8.50×10^{-3}	-	4.20×10^{-2}
Cr	3.00×10^{-3}	3.00×10^{-5}	2.86×10^{-5}	5.01×10^{-1}	2.00×10^{1}	4.20×10^{1}

Table S6. Distribution settings for each parameter in the Monte Carlo simulation [46,54-56]

Parameters	Distribution	Reference
Ring	Log normal	[54]
Rinh	Log normal	[54]
EF	Triangular	[55]
ED	Uniform	[46]
BW	Normal	[54]
AT	Point	[54]
SA	Log normal	[46,54]
AF	Beta	[56]
ABS	Point	[56]
PEF	point	[46]

Table S7. The corresponding target organ toxicity doses for each heavy metal [59]

TTD_i	As	Cd	Cr	Pb
Neurological	0.0003	0.0002	0.01	0.0042
Renal	0.09	0.00083	0.01	0.0006
Cardiovascular	0.0003	0.005	-	0.0013
Hematological	0.0006	0.0008	0.003	0.0013
Testicular	-	0.003	0.005	0.0167
